# Creatine Levels in Patients with Phenylketonuria and Mild Hyperphenylalaninemia: A Pilot Study

**DOI:** 10.3390/life11050425

**Published:** 2021-05-06

**Authors:** Elvira Verduci, Maria Teresa Carbone, Laura Fiori, Claudia Gualdi, Giuseppe Banderali, Claudia Carducci, Vincenzo Leuzzi, Giacomo Biasucci, Gian Vincenzo Zuccotti

**Affiliations:** 1 Department of Pediatrics, Vittore Buzzi Children’s Hospital-University of Milan, Via Lodovico Castelvetro, 32, 20154 Milan, Italy; elvira.verduci@unimi.it (E.V.); laura.fiori@asst-fbf-sacco.it (L.F.); GianVincenzo.Zuccotti@unimi.it (G.V.Z.); 2 Department of Health Science, University of Milan, Via di Rudinì 8, 20142 Milan, Italy; 3 UOS Metabolic and Rare Diseases, AORN Santobono, Via Mario Fiore 6, 80122 Naples, Italy; 4 Pediatric Division, ASST Grande Ospedale Metropolitano Niguarda, Piazza Ospedale Maggiore 3, 20162 Milan, Italy; clodgualdi@yahoo.it; 5 Pediatric Unit, San Paolo Hospital, ASST Santi Paolo e Carlo Hospital, Via di Rudinì 8, 20142 Milan, Italy; giuseppe.banderali@unimi.it; 6 Department of Experimental Medicine, Sapienza University of Rome, Viale Regina Elena 324, 00161 Rome, Italy; claudia.carducci@uniroma1.it; 7 Department of Human Neuroscience, Sapienza University of Rome, Via dei Sabelli 108, 00185 Rome, Italy; vincenzo.leuzzi@uniroma1.it; 8 Department of Pediatrics & Neonatology, Guglielmo da Saliceto Hospital, Via Taverna Giuseppe, 49, 29121 Piacenza, Italy; g.biasucci@ausl.pc.it; 9 Department of Biomedical and Clinical Sciences “L. Sacco”, University of Milan, Via Giovanni Battista Grassi 74, 20157 Milan, Italy

**Keywords:** PKU, mild hyperphenylalaninemia, low-Phe diet, Cr, energy, ATP, physical performance, intellectual performance

## Abstract

Background: Creatine (Cr) levels are strongly dependent on diets, including animal-derived proteins. Cr is an important metabolite as it represents a source of stored energy to support physical performance and potentially sustain positive effects such as improving memory or intelligence. This study was planned to assess Cr levels in PKU children adhering to a diet low in phenylalanine (Phe) content and compared with those of children with mild hyperphenylalaninemia (MHP) on a free diet. Methods: This retrospective pilot study analyzed Cr levels from Guthrie cards in 25 PKU and 35 MHP subjects. Anthropomorphic and nutritional data of the study populations were assessed, compared and correlated. Results: Cr levels of PKU subjects were significantly lower than those of MHP subjects and correlated to the low intake of animal proteins. Although no deficiencies in PKU subjects were identified, PKU subjects were found to have a 26-fold higher risk of displaying Cr levels <25° percentile than MHP counterparts. Conclusions: This pilot study suggests that Cr levels might be concerningly low in PKU children adhering to a low-Phe diet. Confirmatory studies are needed in PKU patients of different age groups to assess Cr levels and the potential benefits on physical and intellectual performance of Cr supplementation.

## 1. Introduction

Creatine (Cr), or methylguanidinoacetic acid, is an amino acid derivative partially obtained by food and partially synthesized de novo by the liver and kidneys from arginine, glycine and methionine. Cr enters the bloodstream to reach target organs, primarily the skeletal muscles which absorb about 95% of the circulating Cr, with the remaining 5% divided among other organs, including brain [1].

Cr uptake occurs by active transport via the deputed Cr transporter SLC6A8 [2], also called CrT1, highly expressed in organs with a high energy requirement (skeletal muscle and brain) or with absorption function (kidney and intestine). In skeletal muscles the enzyme Cr phosphokinase transforms approximately the 2/3 of Cr into phosphocreatine (PCr), used as energy source for ATP synthesis (Figure S1). The remaining third remains in the muscles as free Cr. The total Cr pool (free Cr + PCr) is about 120 mmol/kg of dry muscle mass for a 70 kg individual following a diet including red meat and fish, while vegetarian diets provide a pool (Cr + PCr) about 20–30% lower than non-vegetarians [3,4].

Of the total Cr reserve in an average adult of 70 kg (approximately 120 g), the 1.7% (2 g/day) needs to be replenished daily. In normal conditions 2 g/day of Cr are provided to the organism in equal proportions by endogenous biosynthesis and diet, especially of animal origin. This amount of Cr is converted daily into creatinine and excreted with urine [5]. In addition to providing ATP, the Cr/PCr system also acts as a buffer to delay the depletion of ATP, under conditions of exceptionally high energy demand of the cell [6].

The multifaceted mechanisms by which Cr exerts its beneficial effects include increasing anaerobic energy capacity, decreasing protein breakdown, leading to increased muscle mass and physical performance [7]. In fact, Cr contributes to replenish depleted ATP levels during high-energy demand states-for example intense exercise-or in conditions where energy production is insufficient due to increased (e.g., mental fatigue or some disease states) or impaired (e.g., ischemia, hypoxia) demand [8,9,10,11].

The effects of Cr supplementation have been studied in adolescents performing high energy physical activity (soccer and swimming) to indicate, albeit not definitively, that Cr appears well-tolerated and consistently supporting improvements in swimming and soccer performance [12].

**Figure 1:**
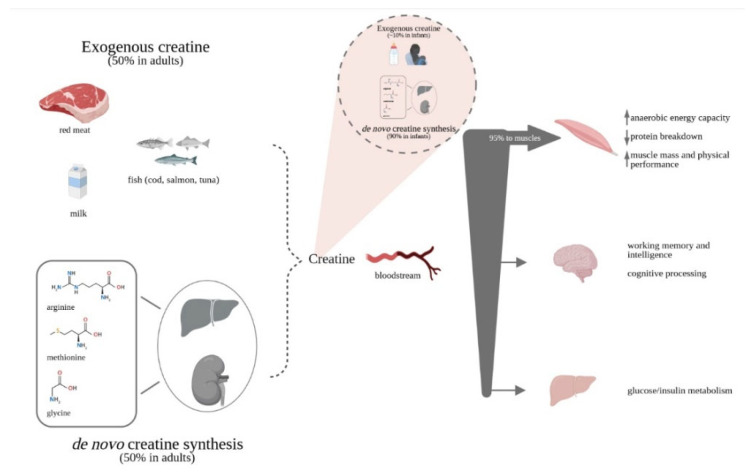
Creatine accrual and potential effects. While in infants the proportions of exogenous and endogenous Cr sources are strongly shifted towards endogenous synthesis, in adults Cr is derived in equal parts from food of animal origin and endogenous synthesis. A vegetarian-like diet, as in PKU, reduces the exogenous sources of Cr both derived from natural foods and often by protein substitutes. A reduced pool of Cr/PCr might affect performance especially in case of high energy demand.

As much as the role of Cr in muscles is well established, other physiological functions of this molecule, such as acting as an antioxidant [13,14], an antiapoptotic [15] and a neuroprotective agent [16,17] have been studied. Albeit not conclusive, there is a wealth of literature indicating the beneficial role of Cr on brain health, specifically concerning cognitive processing [18,19], brain function and recovery from trauma [7]. Cr may be especially helpful in situations of acute stress (i.e., exercise or sleep deprivation) or in chronic pathologic conditions [18,20] (Figure 1). 

In phenylketonuria (PKU), an inherited error of metabolism diagnosed in approximately 1/10.000 newborns in Europe, patients need to follow a life-long low-protein diet specifically tailored to reduce Phe intake and maintain its blood levels under control. PKU subjects’ diet is therefore particularly poor in animal proteins, the major source of dietary Cr. Mild hyperphenylalaninemia (MHP), a milder form of the disorder, is characterized by relatively high levels of Phe in blood but consistently below the threshold level for treatment (360 μmol/L), therefore not requiring a low-Phe dietary regimen to control blood Phe levels.

These two patient populations carry genetic mutations of the Phe hydroxylase (PAH) enzyme but depending on the position of the mutations the phenotype differs. For those who require treatment, the recommended diet closely resembles a strict vegetarian regimen and it is plausible that these patients show lower Cr levels, as in vegetarian subjects [21].

Being consistently on as strict vegetarian diet, which causes significantly lower levels of Cr in blood and in muscles [22], PKU patients may experience a reduced performance being less able to respond to increased energy demand or counteract mental fatigue [23].

Considering the importance of Cr kinase for the maintenance of energy homeostasis in the brain, if this enzyme inhibition also occurs in phenylketonuric patients, it is possible that Cr kinase inhibition may be one of the mechanisms by which Phe is neurotoxic in PKU [24].

A study showed that the administration of Cr or pyruvate can bring benefits in patients with PKU by offsetting brain damage caused by the neurotoxic effects of Phe accumulation [25,26].

In patients with hereditary Cr deficiency, depending on the type of genetic mutation affecting Cr metabolism, different presentations of neurological symptoms are expected. The most common consequences of Cr deficiency are varying grades of intellectual disability and seizures. Other presentations include autistic behaviors, movements and behavior disorders and speech delay [27]. In subjects with deficiencies, long-term high-dose Cr supplementation (0.3–0.8 g/kg/day) is an effective nutritional strategy aimed at increasing brain Cr content [28,29,30,31,32] and producing clinical benefits on cognition, natural development and quality of life [31,33,34].

A few studies showed that the supplementation of Cr may be an effective nutritional strategy to increase PCr content and energy status of the cell in healthy pediatric populations or with specific disorders [35,36,37], but probably not at the same extent as in the adult population [38].

To be noted that, at least in muscles, data suggest that individuals consuming a low-meat diet (who normally show reduced Cr/PCr content) experience greater Cr/PCr accretion following supplementation of this metabolite [39,40]. Additionally, it is important to carefully evaluate a potential age-dependent effect that might be responsible of a different intramuscular Cr uptake by age groups [35].

Unfortunately, the administration of Cr for therapeutic purposes must overcome the obstacle of low cerebral absorption, despite the presence of the carrier, due to the intrinsic characteristics of the blood brain barrier (BBB).

With these premises, we assessed the levels of Cr in school-age children with PKU diagnosis on a Phe-restricted diet and patients of the same age group with MHP who are not required to follow a restricted diet. The objective of the study was to evaluate the consequences of the dietary restrictions on Cr blood levels by comparing results obtained in the two study populations. Correlations between Cr levels and anthropomorphic or nutritional parameters were also assessed.

## 2. Materials and Methods

This retrospective single-center pilot study included school-age children (approximately 6–8 years) followed by the center. 

In particular, study subjects were born between 01/01/2011 to 31/12/2013, had a MHP or PKU diagnosis confirmed by genetic analysis for PAH gene mutation and, in case of PKU diagnosis, were successfully adhering to a low-Phe diet and Phe-free protein substitutes. Protein substitutes taken by study subjects did not contain Cr. No patients in this study received GMP-based protein substitutes, nor sapropterin. 

Successful adherence was considered if patients’ Phe levels resulted below the reference cut-off value of 360 μmol/L, according to the recommendations of the European guidelines [41]. Exclusion criteria included subjects with PKU showing poor compliance to dietary therapy.

Anthropometric data, nutritional intake and Cr values in fasting conditions were evaluated in 25 patients with PKU diagnosis and compared with data of a group of 35 MHP children of comparable age and gender with no dietary restrictions.

The dietary intake of each PKU study subject was recorded by means of a food diary filled out by the parents for three consecutive days (two weekdays and one weekend day). Parents received instructions about how to weight and record food as well as how to report beverage consumption. A dietitian trained the parents to weight and record (before consumption) each food item given to their child, as well as to report weighted leftovers [42]. Quantification and analysis of the energy intake and nutrient composition were performed with an ad hoc PC software (MètaDieta, Me.Te.Da S.r.l., San Benedetto del Tronto, Italy). The dietary assessment presented at the last visit performed before the completion of this retrospective analysis was considered. 

Blood samples for Phe and Cr determination and anthropometric data were taken at the day of the visit.

Cr analysis was carried out on each subject’s dried blood spot (Guthrie card) already available, as obtained for other analyses and reanalyzed to determine Cr blood levels. The samples were maintained at a temperature of −20 °C for optimal preservation. The analyses were performed with a reliable and validated method in tandem mass spectrometry [43].

A power calculation to define the sample size of the study was not performed due to the pilot nature of the study.

Parents/cares of patients gave their consent to the reanalysis.

## Statistical Analysis

Descriptive data are expressed as mean ± standard deviation (DS), median and range of variability and 25°–75° percentile (continuous variables) or number of observations and percentage (discrete variables). The Kolmogorov–Smirnov test was used to test the normality of continuous variables. The comparison of demographic, anthropometric, biochemical data between PKU subjects on or not on diet performed for continuous variables by Student t-test for independent data or Mann–Whitney test, as appropriate. For discrete variables, the chi-squared test or the exact Fisher test or the Mann–Whitney test were used, as appropriate. The association between Cr and other nutritional variables through the Pearson or Spearman correlation coefficient, when appropriate, has been tested.

Values of the statistical significance level p below 0.05 (two-tailed test) were considered statistically significant. Spss version 25.0 (SPSS Inc., Chicago, IL, USA) for Windows (Microsoft, Redmond, WA, USA) was used for statistical analysis.

## 3. Results

The characteristics of the study population are listed in Table 1, divided by disorder diagnosis. The two groups of subjects are balanced as concerns gender ratio and mean age.

Table 2 reports the mean and median anthropometric characteristics of the study population, the corresponding z-scores and the results of the intergroup comparisons. No differences have been identified for any of the measured parameters or the corresponding z-scores between PKU and MHP subjects. 

The nutritional parameters of the study subjects (Table 3) were measured as energy, carbohydrate and lipid intake, protein intake (total, animal-derived and percentage of caloric intake) and Phe intake. In PKU patients, total protein intake corresponds to the sum of proteins from natural foods, Phe-free protein substitutes (PEs) and special low-protein products. Cr levels were also assessed.

Parameters were compared between the PKU and MHP populations. Energy, carbohydrate and fat intake did not significantly differ between PKU and MHP subjects. PKU subjects, coherently with the low-Phe diet they need to follow, show a significantly reduced protein intake. The significantly lower amount of dietary animal-derived proteins, an important source of Cr, could be linked to the significantly lower Cr levels observed in PKU vs. MHP subjects. 

Cr precursors intake was also measured in the two subgroups. Glycine resulted similar in the two subgroups (2.52 ± 0.43 mg/day in PKU subgroup vs. 2.50 ± 0.29 mg/day in MHP subgroup; *p* = 0.91, Student t-test for independent data) while methionine (0.85 ± 0.15 mg/day in PKU patients vs. 1.34 ± 0.15 mg/day in MHP subjects *p* < 0.001, Student t-test for independent data) and arginine (2.24 ± 0.38 mg/day in PKU patients vs. 3.72 ± 0.37 mg/day in MHP subjects; *p* < 0.001, Student t-test for independent data) were significantly less in the diets of patients with PKU than with MHP. 

The low-Phe diet is directly associated with the significantly lower Phe intake in subjects with PKU.

**Figure 2:**
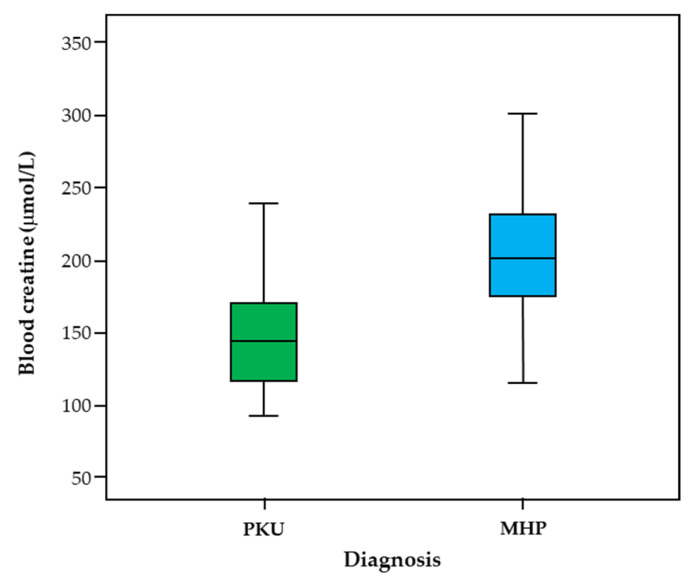
Creatine values in school-age subjects with PKU and MHP.

Figure 2 shows the median Cr values, 25% and 75% quartiles and minimum and maximum Cr values as measured in each subgroup. The distribution of Cr levels in school-age subjects was similar in both groups, but with median values lower for PKU patients than for MHP subjects.

**Figure 3:**
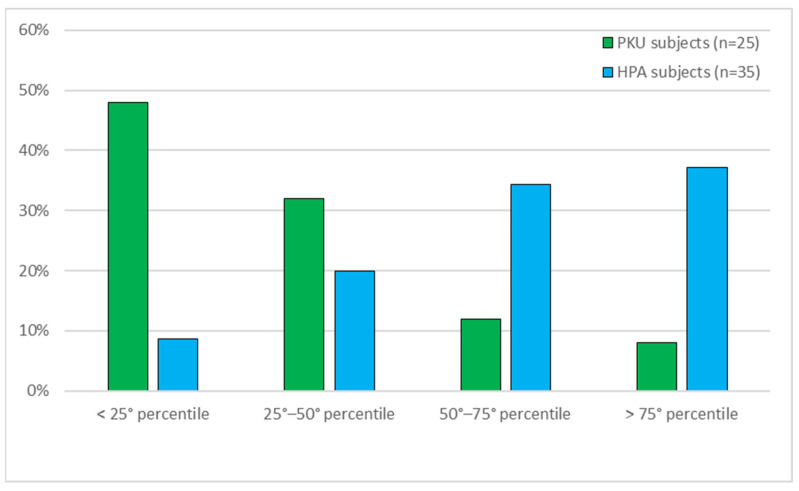
Distribution by quartiles of creatine values in PKU and MHP subjects. 25° percentile: 142.0 µmol/L; 50° percentile: 180.5 µmol/L; 75° percentile: 221.75 µmol/L.

The different distribution of Cr levels was calculated with respect to the breakdown into percentiles of Cr values, for PKU and MHP subjects (Figure 3). A statistical significance of the difference between the two groups of 0.001 was found (Mann–Whitney test). PKU subjects show significantly more subjects with low Cr levels than MHP subjects, whose trend is opposite.

In addition, assuming the Cr values <25° percentile as the reference category, the Odds Ratio (95% confidence interval) for PKU patients compared with MHP subjects is: 7.42 (1.23–45.00) for the 25°–50° percentile, 1.62 (0.23–11.46) for the 50°–75° percentile and 26.0 (3.69–183.42) for the >75° percentile, indicating that PKU subjects have an approximate risk of having Cr values of <25° percentile 26 times higher than MHP subjects.

Correlation coefficients were calculated for all the study subjects (PKU and MHP) to assess if any anthropometric or nutritional parameter could be associated with Cr levels (data not shown). The correlations appear weak for age, BMI (including BMI z-score), energy, carbohydrate and fat intake. Moderate positive correlation with Cr levels is found for all protein intake parameters as well as Phe intake.

## 4. Discussion

Patients with PKU need to follow a life-long low-Phe diet to limit the intake of this essential amino acid and maintain its levels within the recommended ranges for the age. To reach this goal, protein-rich foods, usually of animal origin, are highly limited and compensated by supplementation of Phe-free protein substitutes [41]. Daily Cr requirement in adults is approximately 2 g [2] and is met partly by endogenous Cr synthesis and partly through diet, where food of animal origin accounts for about the 50%. Cr bioavailability depends on intestinal absorption, which has been estimated to be about 80% of the overall Cr intake. A good amount of Cr is in body reservoirs and daily 1.7% of total Cr stored in the body (i.e. the 2 g) needs to be replenished. As the exogenous source of Cr is mainly food of animal origin, particularly red meat or fish (beef: 4.5 g/kg; cod 3 g/kg; salmon 4.5 g/kg; tuna 4 g/kg) and milk to a lesser extent (0.06–0.1 g/L; human milk 0.01 g/L) [44,45], subjects with PKU are at risk of living with suboptimal levels of this important metabolite.

Due to the role of nutrition and dietary habits in growth patterns, anthropometric and nutritional data have been assessed in the study subjects. As no differences were visible between PKU and MHP subjects, it could be supposed that good adherence to the recommended dietary regimen, which is specifically tailored to provide a healthy nutritional intake, supports the same growth patterns of subjects that follow a normal diet.

Despite this, it is always very important to control weight and BMI in PKU patients not only in light of some reports of higher mean weight in PKU patients with respect to the normal population of the same age, especially in critical periods like in infancy and adolescence and particularly in females, but even more importantly to grant the wellbeing of these patients over time [46].

The analysis of the nutritional intake of the study subjects showed a similar energy intake of PKU and MHP subjects, being this parameter well monitored in PKU dietary regimens in order to ensure a correct intake of calories. Obviously, the protein intake was highly different in the two groups. Dietary intake was evaluated by means of a 3-day diary, compiled by the family of each study subject. The reduced protein intake, especially of animal origin (2.40 g/day vs to 32.94 g/day; *p* < 0.001), in subjects with PKU is consistent with the significantly lower Phe intake as well as Cr blood levels. 

In addition, Phe-free protein substitutes, that often do not contain Cr and did not in this particular study and the special low-protein products added to the PKU diet, provide insignificant dietary amounts of Cr. To be also noted that a reduced availability of Cr precursors in this group of patients, represented by a significantly lower intake of methionine and arginine, may have contributed to the observed lower Cr blood levels in the PKU patient population. 

The distribution of Cr levels shows an undeniable diverse distribution of the measured values in subjects with PKU who display Cr levels mostly falling in the <25° and in the 25°–50° percentiles, contrarily to subjects with MHP who mostly display Cr values in the 50°–75° and in the >75° percentiles, making PKU subjects 26 times more likely to have Cr levels <25° percentile than MHP subjects. 

A correlation analysis of the Cr levels of all study subjects and the measured anthropometric or nutritional parameters showed only very weak or weak correlations between Cr levels and anthropomorphic parameters in school-age children. The influence of Cr on muscle mass measured by the BMI index, although not specifically measuring lean mass and other body compositions parameters, does not show a strong correlation.

As expected, correlations become stronger when protein and Phe intake are considered, as Cr levels are also a function of the animal-derived protein intake, as Phe levels are.

Although this study did not reveal Cr deficiencies in the study PKU subjects, the likelihood of suboptimal lower-end Cr levels in these patients should be monitored and considered.

Cr has been studied for its effects in representing a source of ATP to be used, for example, during intense physical exercise or more in general in case of increased demand of energy and lower reserves might result in lesser ability of responding to these energy demands. This could contribute to limit the performance of PKU subjects, especially if they engage in high-energy physical activity. The possibility to supplement Cr in PKU subjects is supposed to exert positive effects, based on results of studies showing a better Cr accrual in vegetarian healthy subjects than in healthy adults consuming meat and fish. Published studies also confirmed that Cr supplementation can compensate the low Cr levels, typically observed in vegetarian subjects. Data in children are limited, but the vegetarian-like diet of PKU subjects theoretically supports a positive effect of Cr supplementation or use of Phe-free amino acid mixtures enriched with Cr also in this population.

Another aspect to consider is the possibility to evaluate Cr levels, already from the first months and years of age. In fact, if in normal newborns and infants receiving human milk or type-1 formula milks the endogenous Cr synthesis compensates the low content of Cr (9% and 36% of the daily recommended intake for Cr for human or formula milk respectively [45]), this might not be the same in patients with PKU. These patients receive only a portion of human milk and the formula milks for PKU do not always contain Cr. The European regulation (UE 2016/127 [47]), for example, does not report information concerning the presence of Cr in milk formulas. As the endogenous Cr synthesis in the growing infant imposes a considerable metabolic burden in terms of amino acid requirements, it could be hypothesized that this might be even stronger in children with PKU receiving formula milk with no or too little Cr content. Prospective clinical trials are needed to determine Cr requirements in newborns and infants with PKU.

Cr supplementation in PKU could also sustain positive effects on cognitive function, although more data are needed to derive conclusions especially considering that data evaluating the increase of Cr or PCr following Cr supplementation are inconclusive. A study clearly reported that Cr supplementation does not seem to increase significantly in the brain [35], likely due to transportation blockade through the BBB, while another study indicated an increased presence of Cr and PCr in the brain when measured by NMR spectroscopy [48]. What is probably reassuring is that indications that memory, intelligence or performance are improved following Cr administration, a positive clinical result. Clearly, the effects of Cr supplementation warrant further evaluation, especially in PKU subjects who might benefit of this supplementation more than the non-vegetarian population.

This study has a few limitations; it is a retrospective study in a relatively small sample, a consequence of being a single-center experience. Being the first study, to our knowledge, to measure this parameter in children with PKU we considered the retrospective pilot study approach reliable enough to give a preliminary overview of this aspect. Further confirmatory studies should be planned to identify if Cr levels are below the average normal values in PKU populations of different age groups, how they correlate with Cr/creatinine urine levels and as a further step, to verify if Cr supplementation provides significant clinical benefits. In addition, any conclusions on the correlation of Cr levels and BMI should be further verified by measuring body fat and the amount and composition of lean mass, important parameters not measured in this study. Moreover, the evaluation of the physical activity levels of the study population could have been an interesting parameter to discuss in light of its potential impact on Cr balance.

## 5. Conclusions

The measurement of Cr blood levels on dried blood spots is an easy, non-invasive and low-cost analysis that can be performed in parallel with the regular Phe level monitoring in PKU patients.

This study clearly identified the increased likelihood for PKU subjects of displaying lower-end levels of Cr than MHP subjects, although generally not below the lower range of values for the normal population [43].

The preclusion of animal-derived proteins, especially meat and fish, in the diet of PKU children may well explain the lower Cr levels with respect to MHP subjects.

Due to the importance of Cr in controlling energy capacity, decrease of protein breakdown, physical performance and potentially exerting other positive effects in the CNS, the results of this study suggest monitoring this parameter in PKU patients in childhood to consider Cr supplementation and warrants evaluations also in the early stages of life.

Confirmatory studies of the clinical benefits of Cr supplementation and the identification of an effective Cr dose to safely obtain positive clinical results in patients with PKU, could reveal important to further tailor the nutritional quality of PKU diets and consequently patients’ wellbeing.

## Figures and Tables

**Table 1:** Characteristics of the subjects with PKU or MHP included in the study.

	PKU Subjects	MHP Subjects
Diagnosis	PKU (Phe ≥ 360 μmol/L)	PKU (Phe < 360 μmol/L)
Subjects (n)	25	35
Dietary treatment	Yes	No
Gender (n, m/f)	13/12	18/17
Mean age (years) ± SD	6.89 0.81	6.82 0.85

**Table 2:** Intergroup comparisons of the anthropometric characteristics of the school-age (Group B) subjects included in the study.

	PKU Subjects (*n* = 25)		MHP Subjects (*n* = 35)		
Anthropometric Parameters	Mean (SD)	Median (Min–Max)	Mean (SD)	Median (Min–Max)	*p* ^†^
Weight (kg)	23.28 (5.16)	24.0 (16.0–35.0)	23.34 (4.50)	23.0 (15.0–35.0)	0.960
Height (cm)	121.28 (7.53)	123.0 (109.0–139.0)	120.54 (6.94)	119.0 (105.0–134.0)	0.697
BMI (kg/m^2^)	15.2 (1.91)	15.0 (12.0–19.0)	15.68 (2.37)	15.0 (13.0–24.0)	0.402
z-score (weight)	0.05 (1.20)	−0.20 (−1.79–2.08)	0.33 (1.24)	0.49 (−1.75–4.42)	0.381
z-score (height)	0.05 (0.98)	−0.12 (−1.53–2.40)	0.13 (1.05)	0.21 (−2.41–1.98)	0.760
z-score (BMI)	0.0 (1.19)	0.32 (−2.45–1.83)	0.28 (1.35)	−0.09 (−1.97–5.16)	0.399

† Statistical significance of the difference between PKU and MHP groups (Student t-test for independent data or Mann Whitney test).

**Table 3:** Differences between dietary regimens in PKU and MHP subjects.

	PKU Subjects (*n* = 25)		MHP Subjects (*n* = 35)		
Nutritional Intake	Mean (SD)	Median (Min–Max)	Mean (SD)	Median (Min-Max)	*p* ^†^
Caloric intake (kcal/day)	1480.52 (291.72)	1442.0 (969.0–2189.0)	1564.68 (182.31)	1576.0 (1002.0–2069.0)	0.105
Carbohydrate intake (g/day)	225.36 (52.30)	227.0 (138.0–343.0)	230.25 (26.03)	234.0 (154.0–284.0)	0.626
Fat intake (g/day)	47.72 (15.26)	46.0 (24.0–81.0)	44.74 (7.56)	44.00 (25.0–71.0)	0.405
Total protein intake (g)	36.88 (6.62)	40.0 (22.0–47.0)	59.45 (7.06)	61.00 (36.0–70.0)	<0.001 *
Animal-derived protein intake (g)	2.40 (2.43)	2.0 (0.0–9.0)	32.94 (5.27)	33.00 (13.0–45.0)	<0.001 *
Protein intake (% of caloric intake)	9.8 (2.62)	10.0 (5.0–17.0)	14.82 (1.27)	15.00 (11.0–18.0)	<0.001 *
Phe intake (mg/day)	594.36 (405.8)	500.0 (141.0–1975.0)	3002.80 (348.36)	3074.0 (1849.0–3539.0)	<0.001 *
Creatine ** (µmol/L)	151.64 (41.27)	145.0 (94.0–241.0)	208.51 (48.39)	202.00 (115.0–322.0)	<0.001 *

† Statistical significance of the difference between PKU and MHP groups (Student t-test for independent data or Mann Whitney test); * Statistically significant difference (*p* < 0.05); ** Cr reference range: 36–344 (149 ± 51) µmol/L [43].

## Supplementary Materials

The following are available online at https://www.mdpi.com/article/10.3390/life11050425/s1, Figure S1: The Creatine/Phosphocreatine system. Cellular Cr is transformed into PCr by mCK. Once transported into the cell cyto-sol, cellular PCr contributes, together with Cr obtained by glycolysis, to the creation of the Cr/PCr pool and correspond-ingly the ATP/ADP pool. Cytosolic CK can utilize PCr stores when energy demand (ATP) is increased.
