# Supplementary material for: Creatine Levels in Patients with Phenylketonuria and Mild Hyperphenylalaninemia: A Pilot Study
Source: Life (Basel). 2021 May 6;11(5):425. doi: 10.3390/life11050425 (PMC8148514; doi:10.3390/life11050425)
Supplement: Supplementary file 1 [file life-11-00425-s001.zip › life-1184473-supplementary.pdf]

life

Supplementary material of Creatine Levels in Patients with Phenylketonuria and Mild Hyperphenylalaninemia: A Pilot Study

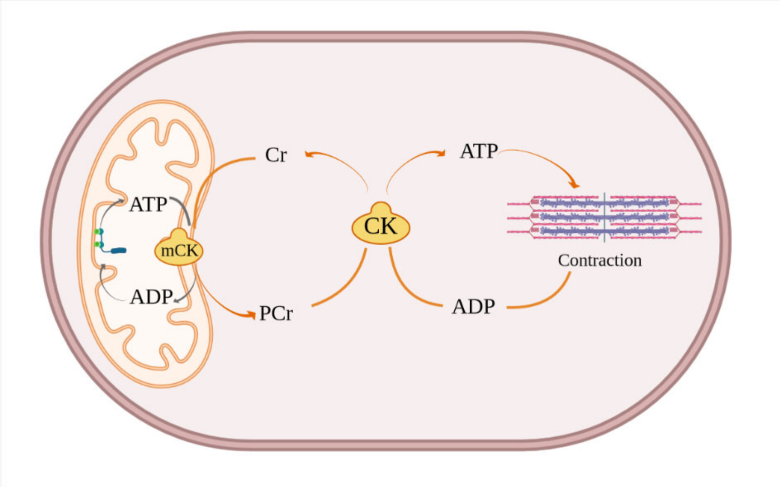

Figure 1. The Creatine/Phosphocreatine system. Cellular Cr is transformed into PCr by mCK. Once transported into the cell cytosol, cellular PCr contributes, together with Cr obtained by glycolysis, to the creation of the Cr/PCr pool and correspondingly the ATP/ADP pool. Cytosolic CK can utilize PCr stores when energy demand (ATP) is increased. Cr and PCr are metabolized into creatinine which, following free diffusion into the bloodstream, is excreted by the kidney. mCK: mitochondrial creatine kinase; CK: creatine kinase; ATP: adenosine triphosphate; ADP: adenosine diphosphate.
